# Aberrant expression of the embryonic transcription factor brachyury in human tumors detected with a novel rabbit monoclonal antibody

**DOI:** 10.18632/oncotarget.3086

**Published:** 2014-12-26

**Authors:** Duane H. Hamilton, Romaine I. Fernando, Jeffrey Schlom, Claudia Palena

**Affiliations:** ^1^ Laboratory of Tumor Immunology and Biology, Center for Cancer Research, National Cancer Institute, National Institutes of Health, Bethesda, Maryland

**Keywords:** Brachyury, EMT, prognosis marker, tumor antigen, monoclonal antibody

## Abstract

The embryonic transcription factor brachyury is overexpressed in a variety of human tumors, including lung, breast, colon and prostate carcinomas, chordomas and hemangioblastomas. In human carcinoma cells, overexpression of brachyury associates with the occurrence of the phenomenon of epithelial-mesenchymal transition (EMT), acquisition of metastatic propensity and resistance to a variety of anti-cancer therapeutics. Brachyury is preferentially expressed in human tumors vs. normal adult tissues, and high levels of this molecule associate with poor prognosis in patients with lung, colon and prostate carcinomas, and in breast cancer patients treated with adjuvant tamoxifen. Brachyury is immunogenic in humans and vaccines against this novel oncotarget are currently undergoing clinical investigation. While our group and others have employed various anti-brachyury antibodies to interrogate the above findings, we report here on the development and thorough characterization of a novel rabbit monoclonal antibody (MAb 54-1) that reacts with distinct high affinity and specificity with human brachyury. MAb 54-1 was successfully used in ELISA, western blot, immunofluorescence and immunohistochemistry assays to evaluate expression of brachyury in various human tumor cell lines and tissues. We propose the use of this antibody to assist in research studies of EMT and in prognostic studies for a range of human tumors.

## INTRODUCTION

Brachyury (also known as “T”) is a member of the T-box family of transcription factors, characterized by a conserved DNA-binding domain called the T-box [1, 2]. In general, T-box transcription factors are vital to the development of the vertebrate embryo as they play key roles in the control of morphogenesis and cell fate decisions [3]. In the mouse, for example, the transcription factor brachyury is essential for the formation and differentiation of the posterior mesoderm, a process that involves a physiological conversion of epithelial cells into mesenchymal cells (called an EMT) [4]. Analyses of the functional targets of brachyury in different species have demonstrated its predominant regulation of genes involved in cell movement, cell shaping and adhesion [5].

Recent work conducted by various research groups has also elucidated additional roles for different members of the T-box family, which are overexpressed in cancer cells [6]. Among the various T-box proteins, high levels of the transcription factor brachyury have been demonstrated in several human tumors, including chordomas [7-9], hemangioblastomas [10, 11] and a range of human carcinomas [12-18], while brachyury is absent in the majority of normal tissues evaluated, with a few exceptions [13]. In chordomas and hemangioblastomas, expression of brachyury is currently being used to resolve their differential diagnosis from other histological mimics [8, 11].

In the case of human carcinomas, brachyury has been shown to regulate epithelial tumor plasticity by promoting epithelial tumor cells to undergo an EMT [14, 19, 20], resulting in the acquisition of metastatic propensity and, as recently demonstrated, acquisition of resistance to multiple therapeutics [21, 22]. In line with this role, the expression of brachyury has been reported to positively correlate with tumor progression in lung [18], breast [16], colon [23] and prostate carcinomas [17], among others, and it has been recently proposed a predominant role for brachyury in triple negative vs. non-triple negative breast tumors [24].

In addition to its crucial role in the biology of various tumors, the brachyury protein is also being exploited as a novel oncotarget for cancer vaccine interventions against various carcinomas and chordomas [25-27]. There are currently two cancer vaccine platforms against the transcription factor brachyury that are being evaluated in clinical trials [28, 29]. While our group and others have previously employed various polyclonal [7, 8, 17] or murine monoclonal [13, 16] antibodies (MAb) to evaluate the expression of brachyury in a variety of human tissues and in human tumor cell lines, we have found that the specificity and/or affinity of those antibodies are relatively low when evaluated in western blot and ELISA assays. In addition, various immunohistochemical studies have now shown that the localization of the brachyury protein varies among tumor types, being exclusively nuclear or cytosolic in chordomas or hemangioblastomas, respectively [8, 10, 11], while detected in the nucleus and/or the cytosol of human carcinoma cells [13, 16, 17]. It is not known whether these discrepancies are due to intrinsic variations of brachyury among the various tumor types, including the existence of various isoforms of the protein, or simply due to the nature of the antibodies employed across the different studies.

In view of the crucial role of brachyury as a diagnostic marker for chordomas and hemangioblastomas, a prognostic marker for a variety of human carcinomas, and as a target for immunotherapies, a novel MAb directed against brachyury, MAb 54-1, has been developed and characterized. We propose the use of this antibody to assist in research studies of the phenomenon of brachyury-mediated EMT and in prognostic studies for a range of human tumors.

## RESULTS

**Figure 1:**
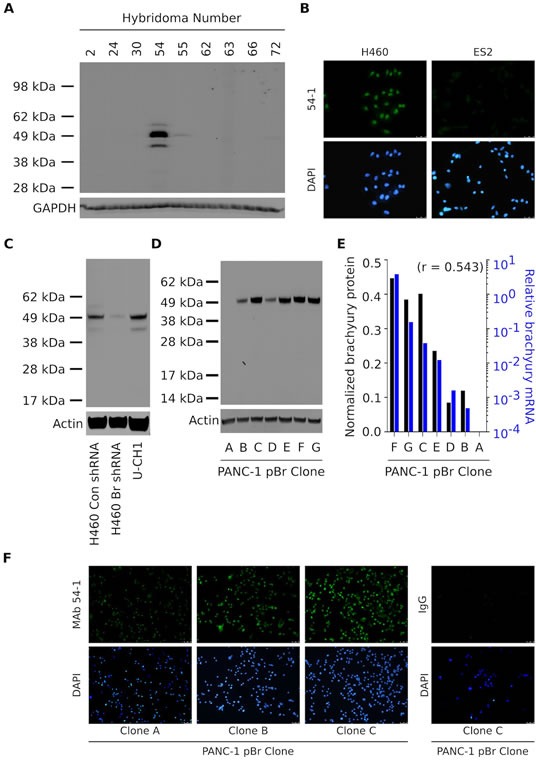
Characterization of a novel anti-brachyury rabbit MAb (A) Western blot of protein lysates from lung H460 cells using culture supernatants from nine hybridomas generated from a rabbit vaccinated with the full-length brachyury protein. (B) Immunofluorescent analysis of the H460 and ES2 cell lines grown in glass coverslips utilizing MAb 54-1 (green signal). Western blot of protein lysates from H460 Con shRNA vs. Br shRNA and chordoma U-CH1 cells (C), and PANC-1 pBr clones (D) with MAb 54-1. (E) Comparison between the levels of actin-normalized brachyury protein detected with MAb 54-1 (black bars) and brachyury mRNA (blue bars) assessed in the same clones by quantitative PCR. Indicated is the Pearson's correlation coefficient. (F) Immunofluorescent analysis of three PANC-1 pBr clones using MAb 54-1 or a control rabbit IgG Ab (green signal).

A novel MAb directed against the T-box transcription factor brachyury was generated as detailed in the “Materials and Methods” section. Following fusion, screening and cloning, culture supernatants from various hybridomas were screened for reactivity against total protein lysates obtained from human lung H460 carcinoma cells, which were previously shown to express high levels of brachyury at the mRNA and protein levels [6, 14, 21]. As shown in Fig. 1A, a hybridoma clone (designated as clone 54) reacted with a 49 KDa protein band corresponding to the expected molecular weight of the brachyury protein. Following subcloning and screening, a resulting hybridoma was expanded and the corresponding rabbit IgG Ab that was able to recognize brachyury, designated hereafter as MAb 54-1, was purified for further characterization. Fig. 1B shows the ability of MAb 54-1 to stain H460 lung cancer cells by indirect immunofluorescence, while no staining was observed with ovarian ES2 cells, which are negative for brachyury mRNA (data not shown).

The specificity of MAb 54-1 was evaluated by western blot analysis of brachyury expression in H460 lung cancer cells that have been stably transfected with either a control, non-targeting shRNA (H460 Con shRNA) or a brachyury-specific shRNA (H460 Br shRNA). As shown in Fig. 1C, reactivity of the MAb 54-1 was abolished in H460 cells silenced for brachyury (Br shRNA), confirming the high specificity of MAb 54-1. Additionally, MAb 54-1 reacted with a predominant band at the expected molecular weight of the brachyury protein expressed at high levels in the chordoma cell line U-CH1. With both tumor cell lines, MAb 54-1 also reacted with an additional band of approximately 45 KDa.

In additional studies, several single-cell derived populations of the human pancreatic PANC-1 cell line stably transfected to overexpress brachyury (designated as PANC-1 pBr clones A-G) were evaluated by western blot analysis with MAb 54-1 (Fig. 1D). A single protein band corresponding to the overexpressed brachyury protein was detected, at levels directly proportional (*r=0.543*) to the levels of brachyury mRNA expressed by each clone (Fig. 1E). Similarly, immunofluorescent detection of brachyury in three PANC-1 pBr clones that expressed brachyury mRNA levels spanning a 3-log range (Fig. 1F, clones A, B and C) showed a proportional staining with MAb 54-1, ratifying the high specificity of the novel MAb 54-1 for detection of brachyury in the context of various tumor cell lines.

**Figure 2:**
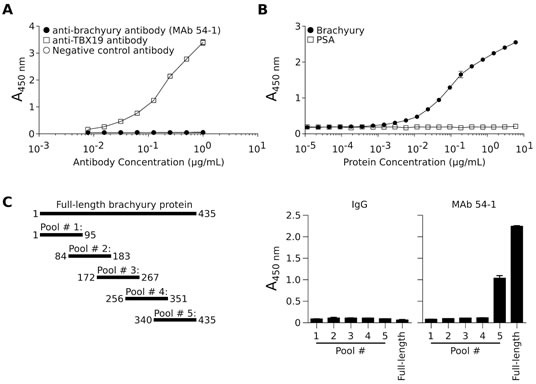
Specificity of the anti-brachyury MAb 54-1 (A) The ability of MAb 54-1 to discriminate between brachyury and the highly homologous TBX19 protein was assessed utilizing a TBX19-specific ELISA assay using MAb 54-1, anti-TBX19, or a control rabbit IgG Ab. (B) MAb 54-1 selectively captures soluble recombinant brachyury protein, but not recombinant PSA protein in an ELISA assay. (C) Schematic representation of each of the peptide pools as compared to the full-length protein, and resulting absorbance obtained when using each peptide pool as the capture protein in an ELISA assay with MAb 54-1.

The brachyury protein shares a high degree of sequence similarity with other members of the T-box family of transcription factors. The closest member of the family, the TBX19 protein, shares an overall 58% amino acid sequence with brachyury and has 91% sequence identity with brachyury in the highly conserved DNA-binding domain. To investigate whether MAb 54-1 is capable of discriminating between brachyury and TBX19, an ELISA assay was performed utilizing recombinant TBX19 protein. As shown in Fig. 2A, MAb 54-1 was unable to recognize TBX19, even when used at high concentrations, in contrast to the positive control, a purified anti-TBX19 Ab that efficiently recognized recombinant TBX19. Furthermore, a capture ELISA assay was conducted with MAb 54-1 bound to 96-wells; as shown in Fig. 2B, MAb 54-1 was efficient at capturing free recombinant His-brachyury protein, but not irrelevant control PSA, with a lower limit of detection at 5.8 ng/mL of recombinant brachyury.

To define the region in which the MAb 54-1 recognizes brachyury, five 15-mer overlapping peptide pools spanning the sequence of brachyury (Fig. 2C) were utilized on an ELISA. As shown (Fig. 2C, right panels), strong reactivity of the MAb 54-1 could be observed only against the peptide pool 5, indicating that the reactive epitope resides within an 84 amino acid fragment located on the C-terminal region of the brachyury protein.

**Figure 3:**
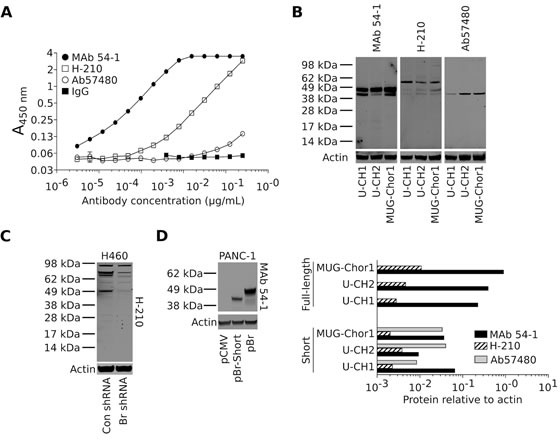
MAb 54-1 binds brachyury with a high affinity (A) Comparison between MAb 54-1 and commercial anti-brachyury antibodies (H-210 and ab57480) by ELISA. (B) Western blot analysis of endogenously expressed brachyury in three chordoma cell lines using the indicated antibodies. *Bottom panels:* Level of actin-normalized protein for each cell line as detected with the indicated antibodies, corresponding to the full length (49 KDa) and the shorter (45 KDa) protein band. (C) Western blot of protein lysates from H460 Con shRNA vs. Br shRNA utilizing the H-210 polyclonal Ab. (D) Western blot of PANC-1 cells expressing a control plasmid (pCMV), a shorter isoform (pBr-Short), or the full-length brachyury protein (pBr) using MAb 54-1.

Nuclear staining of brachyury protein with a commercial, polyclonal rabbit anti-brachyury Ab (H-210) is being currently utilized by many as a tool for the distinction of chordoma tumors from a variety of histological mimics [7, 8, 30]. In addition, a murine MAb (ab57480, purchased from Abcam) has been used for the evaluation of brachyury expression in tumor tissues by immunohistochemistry [13, 16]. Here we have compared the performance of the novel rabbit MAb 54-1 to that of the H-210 and ab57480 antibodies in ELISA and western blot assays. As shown in Fig. 3A, MAb 54-1 exhibited the highest affinity for brachyury when compared to the H-210 and, in particular, the ab57480 murine MAb. On a western blot analysis employing protein lysates prepared from three chordoma cell lines (Fig. 3B) MAb 54-1 was also remarkably efficient at detecting a predominant band corresponding to the expected molecular weight of brachyury (49 KDa) and an additional band of slightly smaller molecular weight (~45 KDa) in all three cell lines. The highly specific performance of the MAb 54-1 contrasted with that of the H-210 Ab that reacted poorly with the 49 KDa band and rendered additional bands of higher molecular weight (Fig. 3B). A quantitative analysis (Fig. 3B, bottom panels) indicated that MAb 54-1 has at least 100-fold higher affinity than the H-210 for detection of the 49 KDa band in western blot. Further evaluation of the H-210 Ab against lysates obtained from H460 cells stably transfected with a control (Con shRNA) vs. a brachyury-specific shRNA (Br shRNA) also revealed several bands that persisted after brachyury silencing, likely representing proteins other than brachyury (Fig. 3C). The murine monoclonal ab57480, though specific, demonstrated binding to the 45 KDa band but showed no reactivity with the full-length brachyury protein in all three chordoma cell lines (Fig. 3B). As several splice variants have been identified for brachyury, we sought to determine if this 45 KDa band could represent a brachyury isoform. A shorter brachyury isoform was cloned from the H460 cell line corresponding to a 377 amino acid protein devoid of 58 amino acids mainly encoded by exon 6 (isoform 2, NM_001270484.1) and subsequently overexpressed in PANC-1 cells. As shown in Fig. 3D, MAb 54-1 was capable of recognizing both the full-length (pBr) and the shorter isoform of brachyury with high efficiency and specificity, thus suggesting that MAb 54-1 is highly specific and has high affinity for at least two isoforms of the human brachyury protein, properties that make it useful for the detection of brachyury expression in multiple types of human tumors.

**Figure 4:**
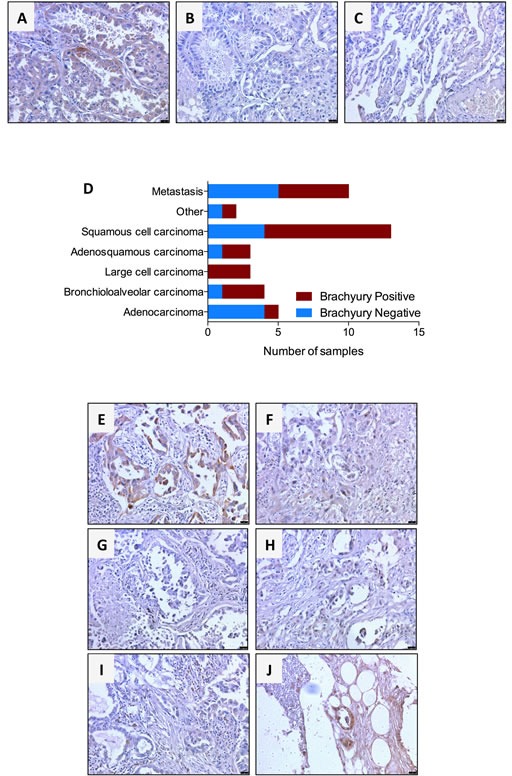
Immunohistochemical detection of brachyury protein in human lung cancers using MAb 54-1 Transmitted light photomicrographs of a primary bronchioloalveolar carcinoma stained with (A) MAb 54-1 versus (B) control isotype IgG. Also shown is a representative staining of normal lung (C) with MAb 54-1. Expression of brachyury was analyzed by immunohistochemistry with MAb 54-1 in 30 cases of primary lung cancer and 10 lung cancer metastases. Shown (D) is the number of brachyury positive and brachyury negative cases for each tumor type. (E-J) Transmitted light photomicrographs of representative primary bronchioloalveolar (E) and large cell (F) primary lung carcinomas. Also shown are matched pairs of primary adenocarcinoma (G) and its corresponding bone metastasis (H) and a primary adenosquamous carcinoma (I) and corresponding matched bone metastasis (J). The brown signal corresponds to brachyury. Magnification 20X, scale bars = 100 μm.

Expression of the transcription factor brachyury has been shown in various tumor types [7, 13, 16, 17, 23] using commercially available anti-brachyury antibodies, prior to the generation of the MAb 54-1. Here, the performance of MAb 54-1 for immunohistochemical detection of brachyury was evaluated against lung tumor tissues. Specificity of the staining with MAb 54-1 was evaluated by comparing its performance to that of a control isotype rabbit IgG and by staining of normal lung tissues. While MAb 54-1 stained lung tumor cells, as shown in Fig. 4C for a representative case of bronchioloalveolar carcinoma, no staining was observed in parallel assessments with the isotype control (Fig. 4B). In addition, MAb 54-1 showed no staining of normal human lung tissues (Fig. 4C). We have also evaluated in parallel the performance of an anti-brachyury Ab (Prestige, available from Sigma-Aldrich), which has been extensively characterized in immunohistochemistry analyses publicly available in the Human Protein Atlas database. Our results demonstrated staining of lung tumor tissues with MAb 54-1 while no staining of the same cases could be observed with the Prestige Ab utilized at the recommended dilution (data not shown). These results were in agreement with those available from the Human Protein Atlas database, where the Prestige Ab shows no reactivity against lung cancer tissues while exhibiting background staining with some normal lung macrophages.

Analysis of 30 primary lung cancer tissues stained with MAb 54-1 revealed an overall positivity for brachyury in 19/30 primary lung tumors, including 1/5 (20%) adenocarcinoma, 3/4 (75%) bronchioloalveolar carcinomas, 3/3 (100%) large cell carcinomas, 2/3 (67%) adenosquamous carcinomas, and 9/13 (69%) squamous cell carcinomas (Fig. 4D). Expression of brachyury was detected either in the nucleus and/or the cytosol of a variable number of tumor cells, ranging from focal to 100% of the tumor cells in each field (Fig. 4A, E, F). In addition to primary tumors, the expression of brachyury was also evaluated in metastatic tissues of lung cancer, including six lymph nodes, two bone metastases, a soft tissue and a small intestine metastasis. Overall, expression of brachyury was seen in 5/10 (50%) metastases including 2/6 lymph nodes, 2/2 bone metastases and a small intestine metastasis (Fig. 4D). Fig. 4G-J shows the results for matched primary and metastatic tumor samples, where a dissociative expression of brachyury can be observed with low expression of brachyury in the primary tumors (Fig. 4G, I) and higher levels of positivity in the corresponding metastatic sites (Fig. 4H, J, respectively).

## DISCUSSION

In light of the growing interest in understanding the role of the phenomenon of EMT in cancer progression, emphasis is currently being placed on comprehensively characterizing the expression and function of molecular drivers of EMT in human tumors [31, 32]. Here we demonstrate the generation and thorough characterization of a novel rabbit MAb (54-1) that binds with high affinity and specificity to brachyury, a driver of human tumor EMT. MAb 54-1 was successfully used in ELISA, western blot, immunofluorescence and immunohistochemistry assays to evaluate the expression of human brachyury protein in various tumor cell lines and tissues.

Brachyury has been originally defined as a tissue-specific transcription factor [1, 2, 33] that is transiently expressed in the vertebrate embryo while being absent in adult tissues. In subsequent studies, brachyury was identified as a gene highly expressed in tumor-derived libraries and rarely observed in normal-tissue derived libraries in the human Unigene database [12]. High expression of brachyury mRNA and/or protein was then reported for a variety of human carcinomas, including lung [13, 18], breast [16], colon [23], prostate [17] and oral squamous carcinomas [34].

The novel rabbit monoclonal MAb 54-1 showed a high level of specificity for at least two isoforms of the brachyury protein, and it was demonstrated here its ability to distinguish between brachyury and the highly related homologous, the TBX19 transcription factor. In accordance with its high specificity, we have also demonstrated there that the region in which MAb 54-1 recognizes brachyury resides within an 84 amino acid fragment in the C-terminus that, notably, has a high degree of divergence between the various members of the T-box family of transcription factors. For example, there is only 31% identity at the amino acid sequence level between brachyury and its closest homologous, the TBX19 protein.

The analysis of lung cancer tissues showed that brachyury is overexpressed in approximately 60% of primary lung carcinomas, in agreement with a previous report [13], as well as in 50% of lung cancer metastases evaluated. Brachyury expression could be detected either in the nucleus and/or the cytosol of the tumor cells; an observation previously reported with other carcinomas [13, 16, 17] and with different sources of anti-brachyury antibodies. Interestingly, the expression of brachyury in chordomas, where mostly 100% of tumors evaluated have shown significantly high levels of brachyury protein, appears to be restricted to the nucleus of the tumor cells. In this regard, nuclear expression of brachyury is currently being used to resolve the differential diagnosis of chordomas from other histological mimics. We therefore believe that staining procedures standardized for the detection of nuclear brachyury in chordomas may need to be adjusted for the detection of variable levels of nuclear and/or cytosolic expression of brachyury in human carcinomas.

Although MAb 54-1 is not intended for use as an immunotherapeutic, brachyury is also been used as a target for the treatment of cancer. It has been previously shown that brachyury is immunogenic in humans [12, 35, 36] and based on those studies, a heat-killed recombinant *Saccharomyces cerevisiae* (yeast) brachyury vaccine and a MVA-poxviral vaccine encoding brachyury and a triad of costimulatory molecules (TRICOM) have been developed and characterized [26] and entered Phase I clinical testing in patients with advanced carcinomas or chordomas [28, 29, 37, 38]. In the context of clinical studies of brachyury-based vaccines, we believe MAb 54-1 could be of potential use to determine what type of tumors express brachyury and could therefore be targets, and to evaluate the presence of brachyury-positive tumor cells pre vs. post-treatment, therefore assisting in the interpretation of the efficacy of brachyury-based therapies.

In conclusion, a parallel assessment of the newly developed MAb 54-1 and two commercially available anti-brachyury antibodies showed the superior performance of the MAb 54-1. While the rabbit polyclonal Ab H-210 demonstrated lack of specificity in western blots and the murine MAb (ab 57480) showed preferential binding to a shorter isoform of brachyury, the MAb 54-1 reacted with high affinity (~100-fold compared to H-210) and marked specificity with both isoforms of brachyury in human tumor cells. There are several potential uses for the newly generated MAb 54-1. Regarding research studies of EMT, MAb 54-1 could be used for the detection of brachyury protein, concurrently with other epithelial and mesenchymal markers, either in human tumor cell lines *in vitro* or *in vivo* growing as xenografts, or in immunohistochemistry studies of primary and/or metastatic tumor lesions. MAb 54-1 could also be used to detect brachyury expression in circulating tumor cells (CTCs) as a means of analyzing features of EMT in CTCs, which has been previously associated with resistance to therapy and disease progression [39]. In addition, MAb 54-1 could be useful to assist in prognostic studies for a range of human tumors, and it will be particularly interesting to compare in future studies the performance of MAb 54-1 to that of other commercially available anti-brachyury antibodies previously utilized in prognostic evaluations.

## MATERIALS AND METHODS

### Cell culture

The human lung H460, ovarian ES2, pancreatic PANC-1, and chordoma U-CH1, U-CH2, and MUG-Chor1 carcinoma cell lines were obtained from American Type Culture Collection (ATCC) and maintained in culture as recommended by the ATCC. All cell lines were newly purchased or their identity confirmed by STR analysis (PANC-1 and H460 cells). Brachyury overexpression and silencing vectors and transfection strategies were previously described [14].

### Rabbit MAb generation

A recombinant His6-tagged, full-length human brachyury protein was produced via a baculovirus expression system in insect cells. This protein was utilized as an immunizing antigen at Epitomics (Abcam). Rabbit immunizations, serum collection, hybridoma fusions and screening, hybridoma cloning and subcloning, and subsequent expansion and IgG purification were conducted by Epitomics, Inc., utilizing their proprietary technology (www.epitomics.com).

### Western blot

Protein lysates from H460, PANC-1 and chordoma cells were prepared with RIPA buffer (Cell Signaling Technology) supplemented with 1 mM Phenylmethanesulfonyl fluoride (Sigma-Aldrich); five to 10 μg of protein was run in each lane. The following primary antibodies against brachyury were used: monoclonal rabbit (MAb 54-1, 1 μg/ml), monoclonal murine ab57480 (purchased from Abcam, 1 μg/ml), and polyclonal rabbit H-210 (purchased from Santa Cruz Biotechnology, Inc; 1/200 dilution). IRDye-800CW conjugated goat anti-mouse or anti-rabbit secondary antibodies (LI-COR Biosciences) were utilized at a 1:5000 dilution. Hybridoma supernatants were screened at a 1:500 dilution. All western blots were imaged and quantified using the Odyssey Infrared imaging system (LI-COR Biosciences).

### Real-time PCR analysis of gene expression

Analysis of brachyury mRNA expression was performed as previously described [14].

### Immunofluorescence and immunohistochemistry

For indirect immunofluorescent detection of brachyury protein, cells were grown on glass coverslips, fixed with 3% paraformaldehyde for 10 minutes at room temperature, permeabilized with PBS containing 0.05% Triton X-100 for 20 minutes at room temperature, and subsequently blocked using PBS containing 1% BSA (CELL Applications, Inc.) and 10% goat sera (Invitrogen Life Technologies). Rabbit anti-brachyury MAb 54-1 was added at 1 μg/ml in PBS + 1% BSA overnight at 4° C. An Alexa-Fluor-488 labeled goat anti-rabbit IgG (Invitrogen Life Technologies) was used at 1:250 dilution in PBS + 1% BSA for one hour at room temperature. Coverslips were stained with DAPI (Invitrogen Life Technologies), and mounted utilizing fluorogel with Tris buffer (Electron Microscopy Sciences). Images were captured utilizing a Leica fluorescent microscope.

Formalin-fixed tumor tissue arrays were purchased from US Biomax, Inc. Tissue sections were deparaffinized in xylene, rehydrated in a series of graded ethanol, and treated with 0.3% H_2_O_2_ in methanol to block endogenous peroxidase activity. Microwave-citrate buffer antigen retrieval method was performed to unmask the antigen. Tissue sections were blocked in horse serum (Invitrogen) for 30 minutes at room temperature and then incubated for 90 minutes at room temperature with MAb 54-1 at 2.4 μg/ml. In addition, an IgG rabbit negative control Ab (Abcam) was used to verify accurate staining method. The ImmPRESS HRP-labeled universal Ab (anti-mouse/anti-rabbit peroxidase polymer detection kit, Vector Laboratories) was used as per the manufacturer's recommendations. Color was developed with DAB peroxidase substrate (Vector Laboratories). Sections were counterstained with haematoxylin. Images were captured utilizing a Leica microscope. Cytosolic and nuclear staining was assessed when determining brachyury positive samples.

### Enzyme-Linked Immunosorbent Assay (ELISA)

Immulon 4 HBX flat-bottom 96-well plates (Thermo Scientific) were coated overnight at 4° C with 50 ng TBX19 protein (Origene Technologies) in PBS and blocked with PBS supplemented with 5% BSA (Cell Applications Inc.). Primary antibodies against TBX19 (GeneTex, Inc.), brachyury (MAb 54-1; H-210, Santa Cruz Biotechnology Inc; ab57480, Abcam) or a control rabbit IgG (Abcam) were added at indicated concentrations in PBS supplemented with 1% BSA (Cell Applications, Inc.) for 1 hour at room temperature. HRP-labeled anti-rabbit IgG (Promega) or anti-mouse IgG (BD Biosciences) secondary antibodies were added at a 1:3000 dilution for 45 minutes at room temperature. The SureBlue TMB microwell peroxidase substrate and stop solutions were used (Kirkegaard & Perry Laboratories, Inc.). Absorbance was measured at 450 nm utilizing a Synergy HT plate reader (BioTek Instruments, Inc.). To map the region recognized by MAb 54-1, five pools of overlapping 15-mer peptides spanning the 435 amino acid sequence of brachyury were used (JPT Peptide Technologies). Plates were coated with 100 ng/well of each peptide pool or a purified recombinant full-length His-brachyury protein.

For antibody capture experiments, plates were coated with 1 μg/ml MAb 54-1 for 3 hours at room temperature; purified His-brachyury or prostate-specific antigen (PSA) protein were added at indicated concentrations in PBS containing 1% BSA, following by overnight incubation at 4° C. Goat anti-brachyury polyclonal Ab (R&D Systems) was added at 1 μg/ml and plates were incubated overnight at 4° C. An HRP-labeled rabbit-anti goat Ab (Invitrogen Life Technologies) was added at 1:3000 dilution for 45 minutes at room temperature. The positive cut-off value for each assay was defined as the antibody or protein concentration that rendered twice the absorbance (450 nm) observed with conjugate blank control wells.
